# Endoscopic ultrasound–guided radiofrequency ablation for management of bleeding gastrointestinal tract malignancies: a report of 2 cases

**DOI:** 10.1016/j.vgie.2026.04.043

**Published:** 2026-04-29

**Authors:** Muhammad Ahmad Bashir, Dayyan Adoor, Vishali Moond, Ayowumi Adekolu, Shyam Thakkar, Shailendra Singh

**Affiliations:** 1 Department of Internal Medicine, West Virginia University, Morgantown, West Virginia, USA; 2 Department of Digestive Diseases, West Virginia University, Morgantown, West Virginia, USA

## Abstract

**Background and Aims:**

Bleeding from advanced gastrointestinal malignancies is often recurrent and difficult to control with conventional endoscopic hemostatic techniques. Endoscopic ultrasound–guided radiofrequency ablation (EUS-RFA) enables targeted ablation of deep tumor tissue and associated vasculature. We describe the use of EUS-RFA as a hemostatic strategy in patients with malignant gastrointestinal bleeding arising from distinct tumor morphologies.

**Methods:**

Two patients with active gastrointestinal bleeding from advanced malignancies underwent EUS-RFA. Case 1 involved a friable, infiltrative duodenal mass, and case 2 involved a large pedunculated gastroesophageal junction tumor. Endoscopic ultrasound was used to delineate tumor depth and vascularity. A 19-gauge EUS-RFA needle was advanced into the tumor, and radiofrequency energy was delivered at 20 W until impedance rise or time limit was reached. Adjunctive argon plasma coagulation was used in 1 case for residual superficial oozing.

**Results:**

EUS-RFA was technically successful in both cases, achieving immediate hemostasis without procedural adverse events. Follow-up endoscopy confirmed no evidence of recurrent bleeding.

**Conclusions:**

EUS-RFA is a feasible and safe endoscopic modality for malignant gastrointestinal bleeding that, unlike traditional hemostatic therapies, provides deep coagulation of tumor tissue and its feeding vessels, serving as a potential hemostatic option for such masses.

## Introduction

Hemorrhage from advanced gastrointestinal malignancies represents a challenging clinical problem, with high rates of recurrence using conventional endoscopic hemostatic techniques. Tumor bleeding is driven by extensive, friable neovascularity and local coagulopathy, often resulting in poor long-term control with clips, injection, or topical therapies alone.^1^ Endoscopic ultrasound–guided radiofrequency ablation (EUS-RFA) enables targeted thermocoagulative necrosis of deep tumor tissue and feeding vasculature under real-time imaging. Although most published experience with EUS-RFA has focused on pancreatic lesions and neuroendocrine tumors, evidence supports its feasibility, safety, and expanding clinical applications in malignant lesions.^2^^,^^3^ Although its use in this context is currently off-label, the EUS-RFA equipment and technique have been previously well described.^4^ Here, we describe 2 cases with distinct tumor morphologies—a friable infiltrative duodenal mass and a large pedunculated gastroesophageal junction lesion, in which EUS-RFA was used as a potential hemostatic option for such masses to achieve control of malignant gastrointestinal bleeding.

## Procedure description

### Case 1

An 84-year-old man with stage IV metastatic pancreatic adenocarcinoma complicated by portal vein thrombosis and superior vena cava syndrome, maintained on therapeutic apixaban, presented with acute on chronic anemia and overt gastrointestinal bleeding. Upper endoscopy revealed a friable, fungating malignant mass in the first portion of the duodenum with active hemorrhage (Fig. 1). Given the high likelihood of recurrent bleeding with standard endoscopic therapy, endoscopic ultrasound (EUS)-guided intervention was pursued using a specialized EUS-RFA needle (Fig. 2) and is detailed in Video 1, available online at www.videogie.org.

**Figure 1:**
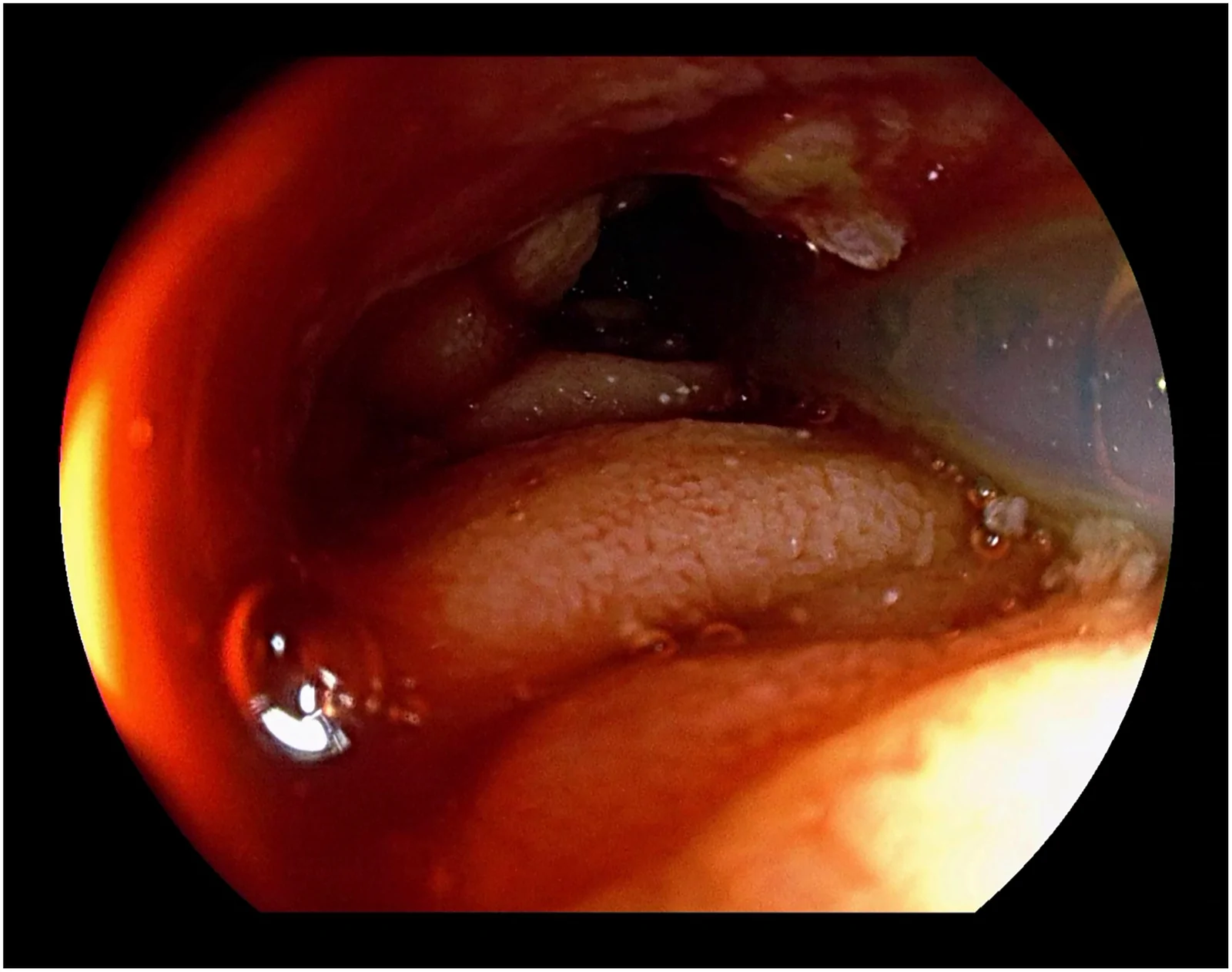
Upper endoscopy demonstrating a friable, fungating malignant mass in the first portion of the duodenum with active hemorrhage.

**Figure 2:**
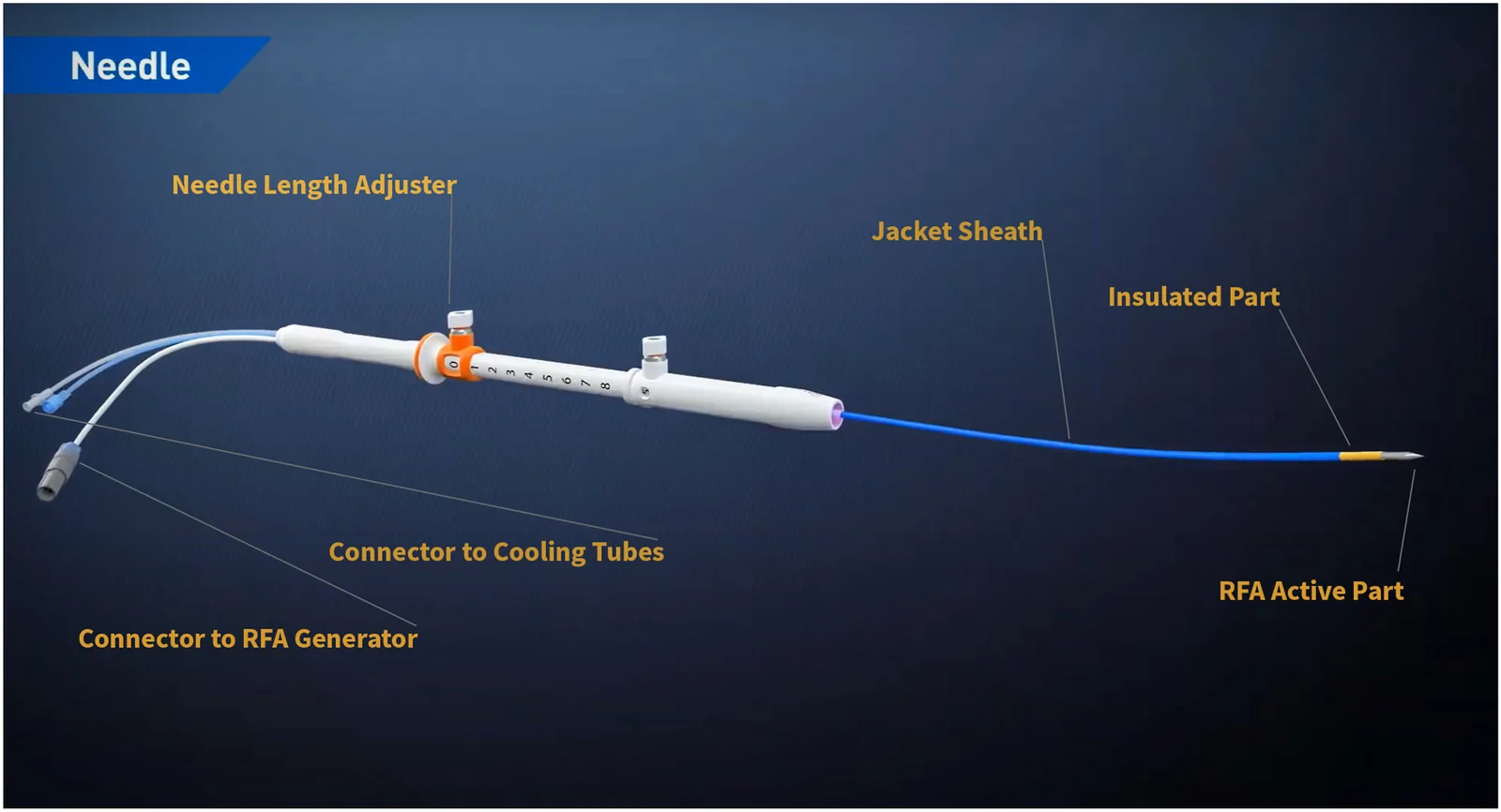
Sample endoscopic ultrasound–guided radiofrequency ablation (RFA) needle used for targeted ablation of malignant gastrointestinal tumors.

Linear EUS delineated tumor depth and vascularity. A 19-gauge radiofrequency ablation (RFA) needle was advanced transduodenally into the neoplastic tissue, with ablation visualized as hyperechoic signal on EUS (Fig. 3). Radiofrequency energy was delivered at 20 W, with each application terminated by a 40-second timeout or an impedance rise. Ten ablation applications were performed, with post-treatment visualization on conventional EGD (Fig. 4). Follow-up endoscopy 1 week later demonstrated a necrotic-appearing lesion with complete resolution of bleeding (Fig. 5). The patient subsequently had adverse events from a peritoneal drainage catheter placed for malignant ascites and opted for hospice care on day 10 after the procedure.

**Figure 3:**
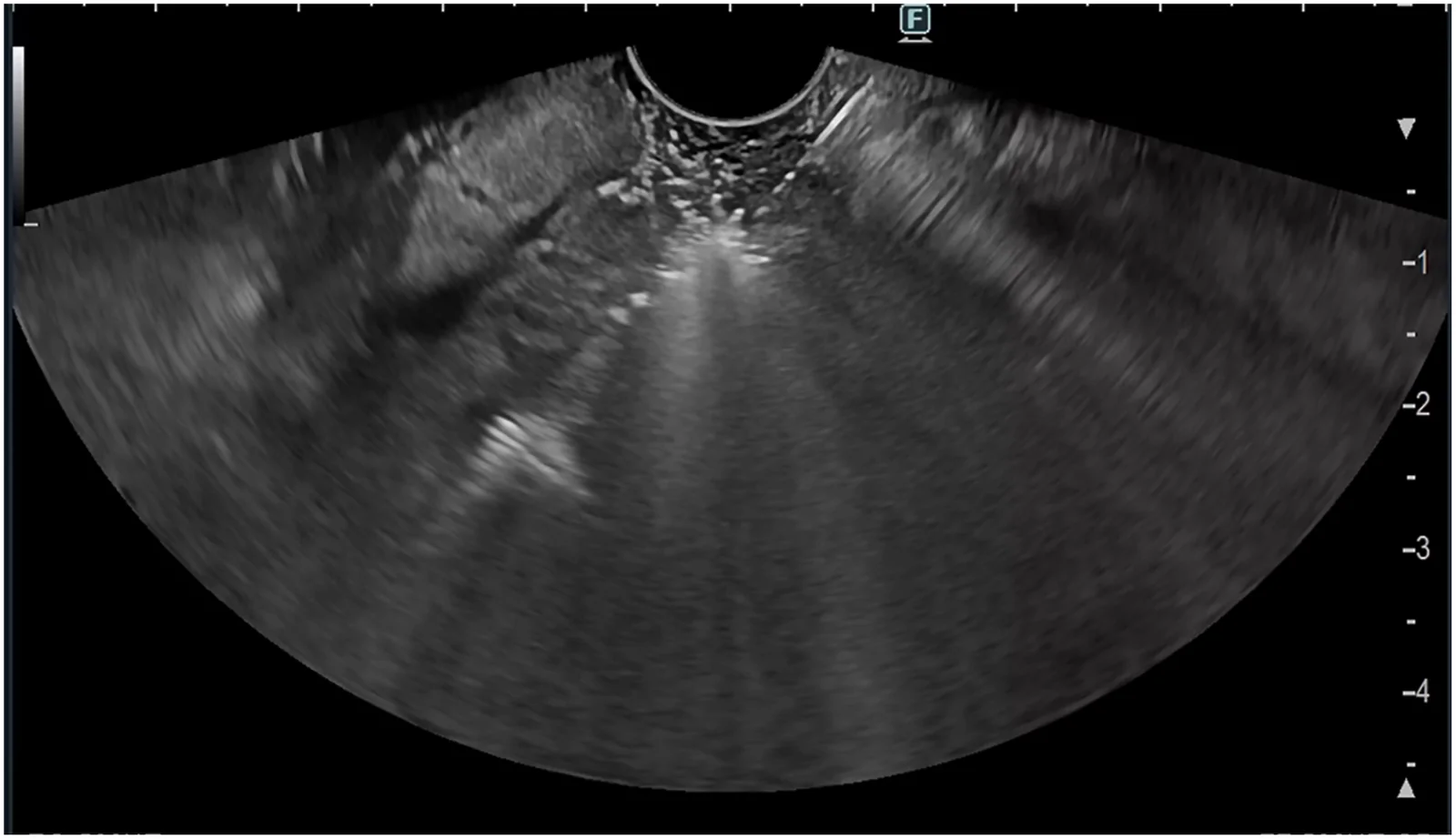
Endoscopic ultrasound view showing the radiofrequency ablation needle positioned within the duodenal tumor, with hyperechoic changes consistent with active tissue ablation.

**Figure 4:**
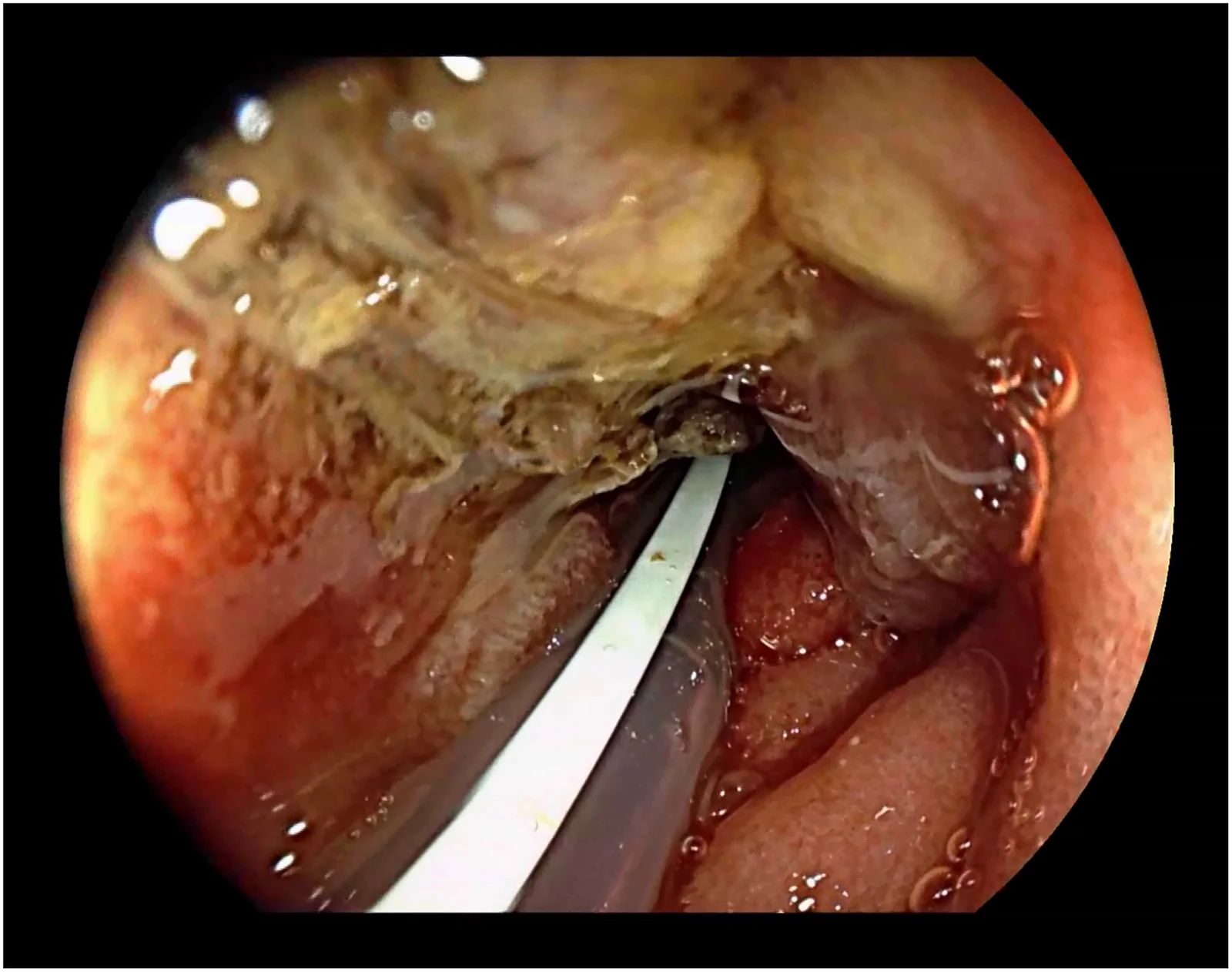
Endoscopic appearance of the duodenal mass immediately following endoscopic ultrasound–guided radiofrequency ablation, demonstrating treated tissue without active bleeding.

**Figure 5:**
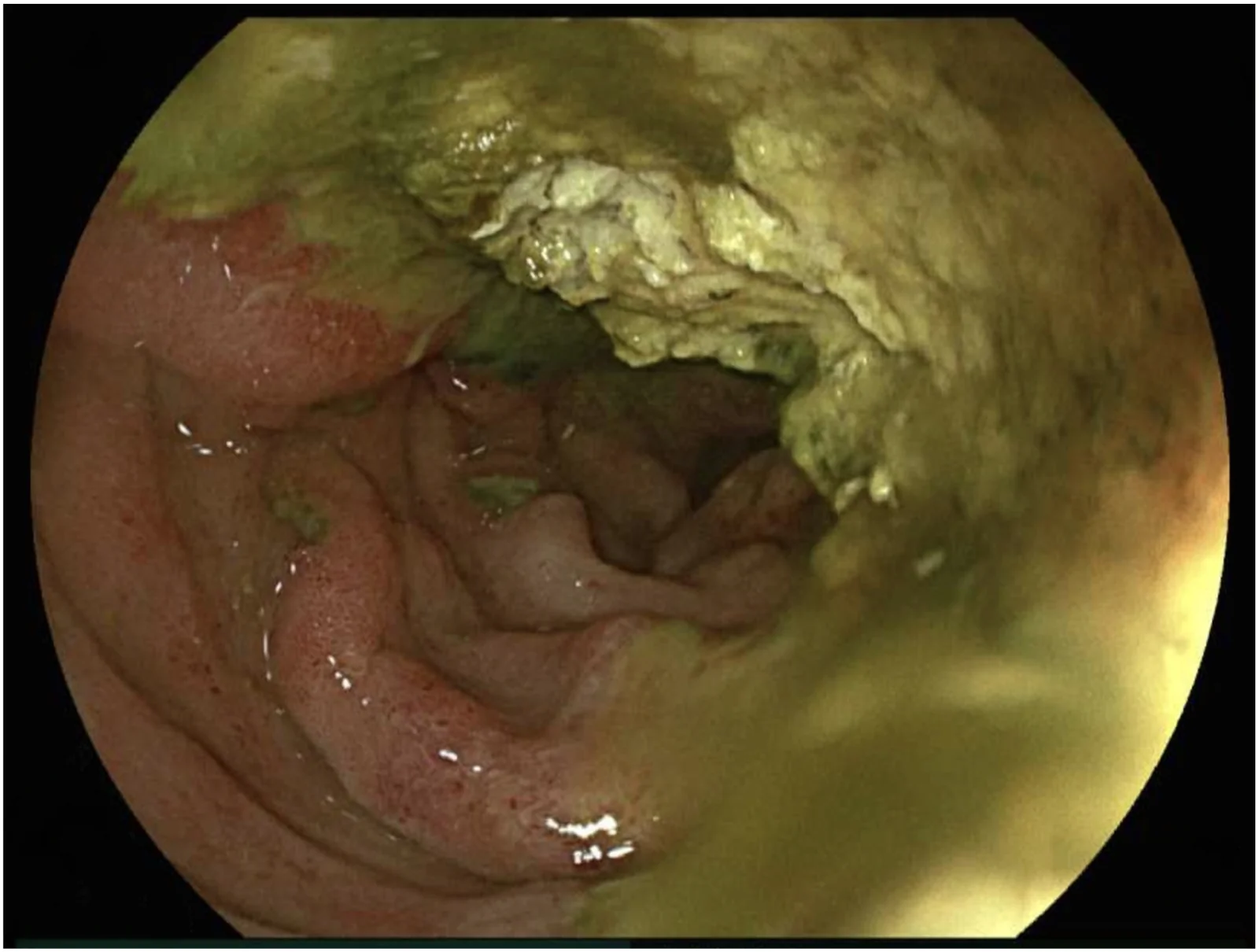
Follow-up endoscopy 1 week after endoscopic ultrasound–guided radiofrequency ablation showing a necrotic-appearing duodenal lesion with complete resolution of hemorrhage.

### Case 2

A 64-year-old woman with chronic kidney disease, diabetes mellitus, epilepsy, hyperlipidemia, and chronic antithrombotic therapy presented with acute on chronic anemia, coffee-ground nasogastric output, and progressive dysphagia. Following unsuccessful hemostasis with spray and injection at an outside hospital, the patient was transferred to our facility for further intervention. Upper endoscopy revealed a large, pedunculated intraluminal mass at the gastroesophageal junction with high-risk stigmata of bleeding (Fig. 6).

**Figure 6:**
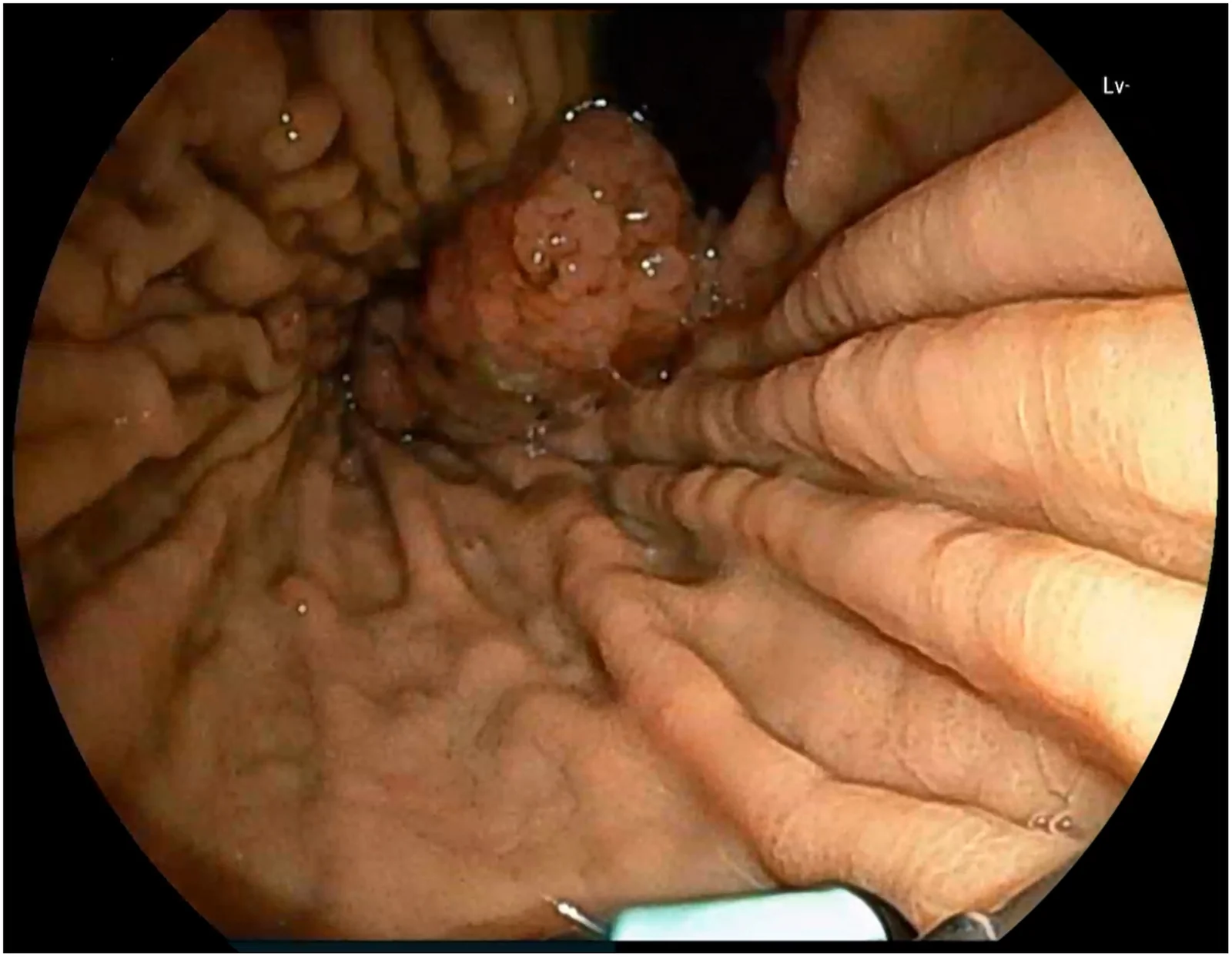
Upper endoscopy revealing a large, pedunculated intraluminal mass at the gastroesophageal junction with high-risk stigmata of bleeding.

EUS-RFA was performed via a transgastric approach. Water immersion enhanced acoustic coupling and visualization. The RFA needle was advanced into the tumor bulk under EUS guidance (Fig. 7), and radiofrequency energy was delivered at 20 W over 34 ablation applications. After deep tumor ablation, adjunctive argon plasma coagulation (APC) was applied to control residual superficial oozing. Post-RFA endoscopic evaluation confirmed immediate hemostasis (Fig. 8). The patient remained hospitalized for 2 weeks after the procedure with no further bleeding noted. The patient was subsequently lost to follow-up after discharge.

**Figure 7:**
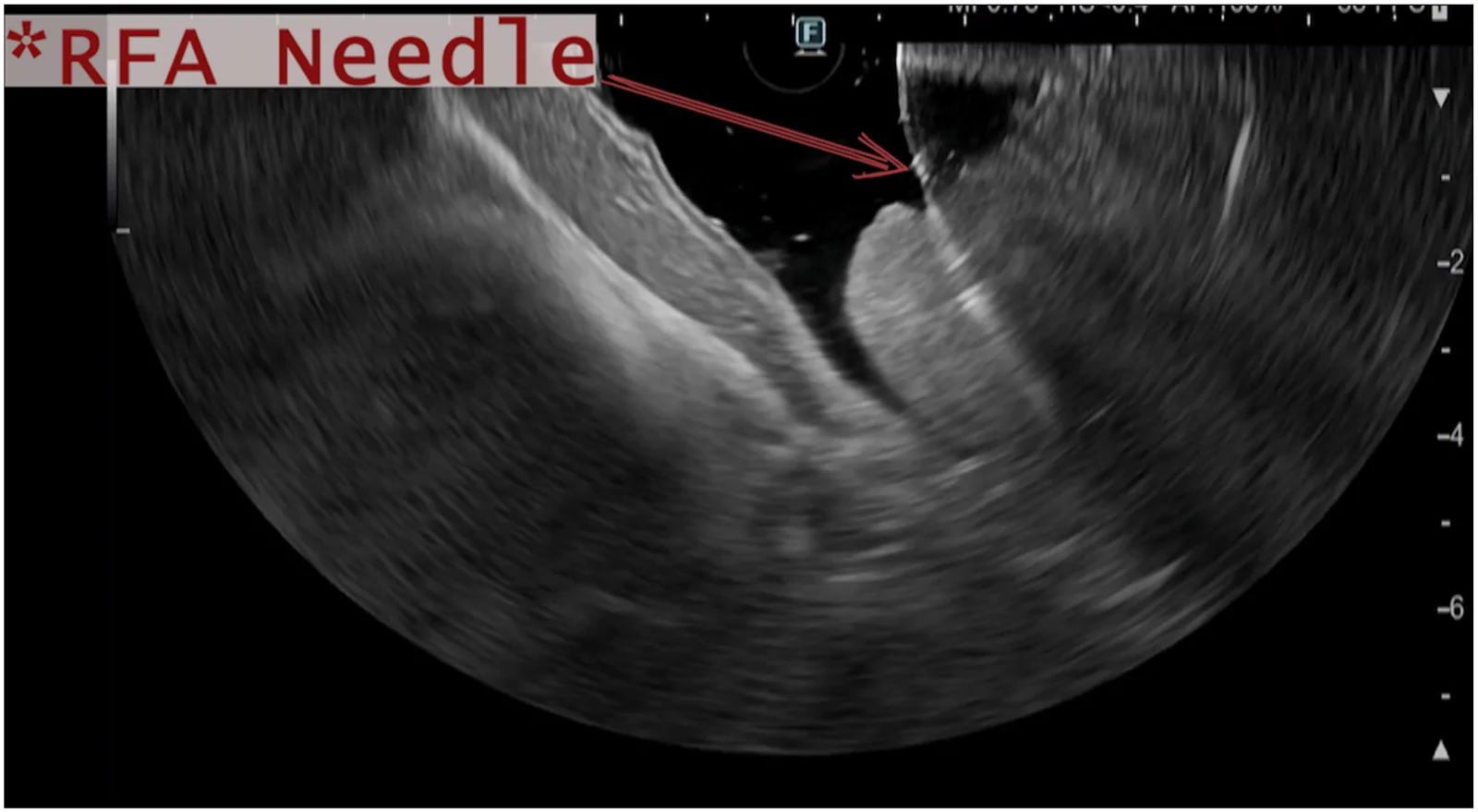
Endoscopic ultrasound image demonstrating transgastric advancement of the radiofrequency ablation (RFA) needle into the gastroesophageal junction tumor during ablation.

**Figure 8:**
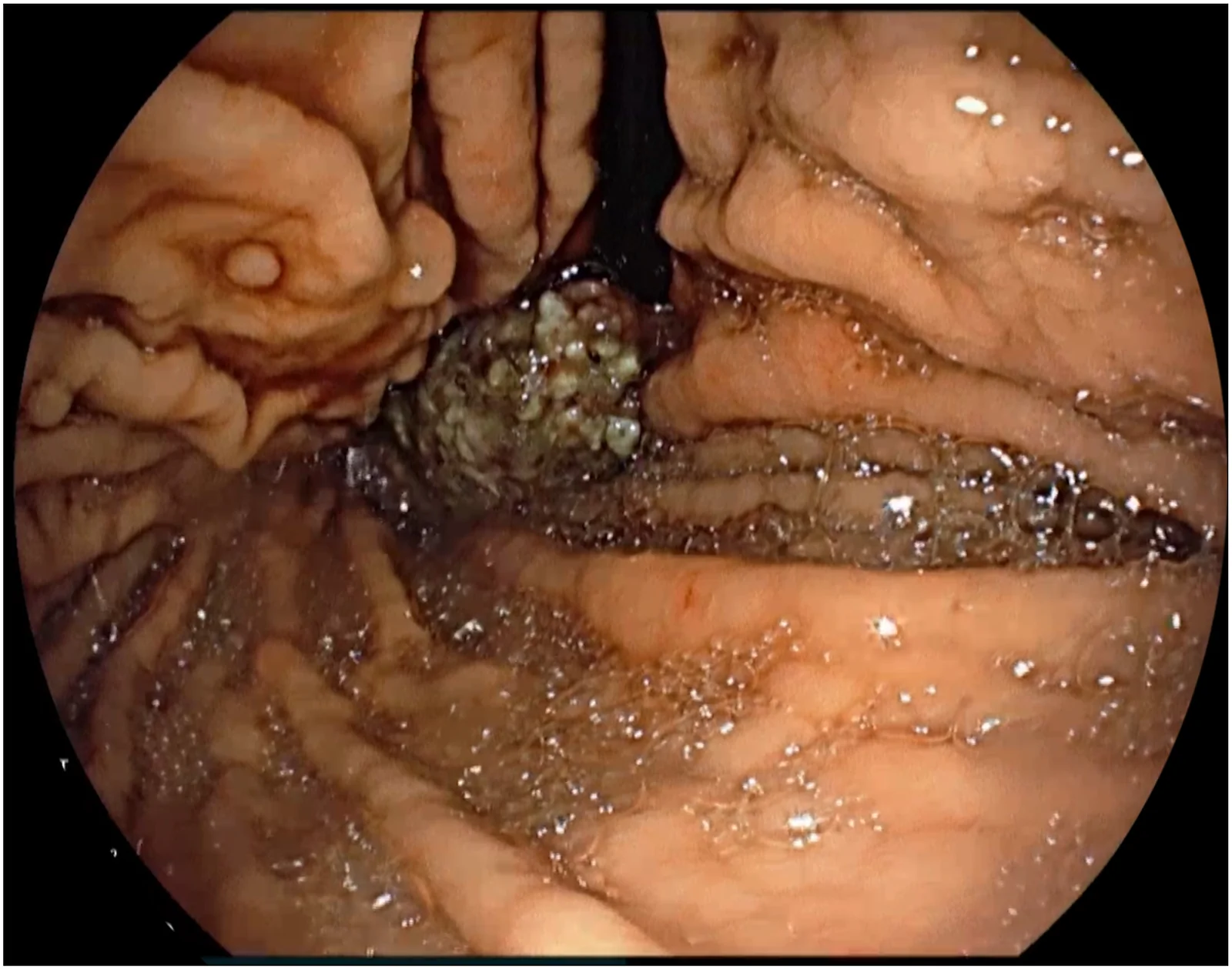
Postablation endoscopic view of the gastroesophageal junction tumor showing effective hemostasis following endoscopic ultrasound–guided radiofrequency ablation and adjunctive argon plasma coagulation.

## Outcomes and discussion

Both procedures were technically successful and well tolerated, with no immediate adverse events, including perforation, uncontrolled hemorrhage, or postprocedural pancreatitis. In case 1, follow-up endoscopy demonstrated complete resolution of bleeding. In case 2, immediate hemostasis allowed the safe resumption of therapeutic anticoagulation with enoxaparin and clopidogrel without recurrent gastrointestinal bleeding during hospitalization.

These cases illustrate the potential utility of EUS-RFA as a hemostatic tool in malignant gastrointestinal bleeding, complementing existing therapeutic modalities. EUS-RFA has been shown to provide targeted thermal ablation with acceptable safety profiles in pancreatic and other solid gastrointestinal tumors, with a growing body of literature describing high technical success and favorable clinical outcomes.^2^^,^^5^ Although most evidence to date pertains to tumor control rather than hemostasis, we hypothesize that the principle of deep coagulative necrosis with destruction of tumor microvasculature may lead to sustained bleeding control.

Prospective studies are needed to define optimal energy settings, procedural protocols, and long-term outcomes. Given the inherent difficulties in managing tumor-related bleeding with conventional endoscopic hemostatic methods, novel approaches such as EUS-RFA merit further investigation as adjunctive or potential hemostatic options for such masses in selected patients with malignant hemorrhage.

## Disclosures

All authors disclosed no financial relationships.

## Supplementary data

Video 1 Endoscopic ultrasound-guided radiofrequency ablation (EUS-RFA) technique and clinical outcomes for the management of active hemorrhage in advanced gastrointestinal tract malignancies.
